# Dataset of process-structure-property feature relationships for AlSi10Mg material fabricated using laser powder bed fusion additive manufacturing

**DOI:** 10.1016/j.dib.2024.110130

**Published:** 2024-02-01

**Authors:** Qixiang Luo, Nancy Huang, Tianyi Fu, Jinying Wang, Dean L. Bartles, Timothy W. Simpson, Allison M. Beese

**Affiliations:** a Department of Materials Science and Engineering, Pennsylvania State University, University Park, PA 16802, USA; b Department of Chemistry, Pennsylvania State University, University Park, PA 16802, USA; c Manufacturing Technology Deployment Group, Inc., Clearwater, FL 33762, USA; d Department of Industrial and Manufacturing Engineering, Pennsylvania State University, University Park, PA 16802, USA; e Department of Mechanical Engineering, Pennsylvania State University, University Park, PA 16802, USA

**Keywords:** Additive manufacturing, Laser powder bed fusion, Processing parameters, X-ray computed tomography, Microstructure, Process-structure-property relationships

## Abstract

This dataset reports microstructure and mechanical property features of AlSi10Mg manufactured using laser powder bed fusion over a wide range of processing conditions. Samples were fabricated with different combinations of laser power, scan speed, and hatch spacing to probe dense regimes as well as porous samples resulting from keyholing and lack of fusion. Pore and grain/sub-grain features for each processing set were quantified. Sample porosity was measured using Archimedes density measurements and X-ray computed tomography (XCT). XCT was also used to characterize the surface roughness of samples along with pore size and morphology. Electron backscatter diffraction (EBSD) was used to characterize the grain size and morphology while scanning electron microscope (SEM) imaging and was used to measure solidification cell size. Uniaxial tension tests were performed to ascertain yield and ultimate tensile strengths, elongation, and elastic modulus, and microhardness was measured using Vickers indentation.

Specifications Table

**Table:** 

Subject	Manufacturing Engineering.
Specific subject area	Dataset with quantified processing, structure (microstructure, defect structure, surface roughness), and mechanical properties for AlSi10Mg manufactured by laser powder bed fusion additive manufacturing [1].
Type of data	Tables.
How the data were acquired	Specimen fabrication: laser powder bed fusion metal additive manufacturing system (ProX DMP 320, 3D Systems, Inc.). Defect pore characterization: X-ray computed tomography (XCT, Phoenix v|tome|x L300 nano/micro-CT system, General Electric) with image processing software (Amira-Avizo 2020.2, Thermo Fisher Scientific and MATLAB v. R2018b, The MathWorks, Inc.); Archimedes porosity measurements (OHAUS EX623). Grain structure characterization: electron backscatter diffraction (EBSD) imaging software (AZtec, Oxford Instruments) with imaging processing software (MATLAB v. R2018b, The MathWorks, Inc.). Sub-grain structure characterization: scanning electron microscope (SEM, Verios G4 XHR, Thermo Fisher Scientific) imaging with imaging processing software (MATLAB v. R2018b, The MathWorks, Inc.). Mechanical testing: Vickers microhardness indentation (LECO LM 110AT); uniaxial tension testing (MTS Criterion Model 45) with digital image correlation using a digital camera (GRAS-50S5M-C, Point Grey Research, Inc.) with analysis software (VIC-2D 6, Correlated Solutions, Inc.).
Data format	Raw and analyzed.
Description of data collection	Uniaxial tension specimens were fabricated using 60 different processing parameter combinations with variations in laser power, scan speed, and hatch spacing to investigate processing-dependent microstructure and mechanical properties. Pore structures were measured via XCT analysis, and grain/sub-grain structures were imaged and analyzed via SEM/EBSD images. Mechanical properties were obtained using uniaxial tension tests and Vickers microhardness measurements.
Data source location	Specimen fabrication: 3D Systems, 700 Marine Dr, Rock Hill, SC 29730, USA; CIMP-3D, 230 Innovation Boulevard, Pennsylvania State University, Innovation Park, PA, USA. Defect pore characterization: Center for Quantitative Imaging (CQI), Pennsylvania State University, University Park, PA, USA. Grain/sub-grain structure characterization: Materials Characterization Lab, Pennsylvania State University, University Park, PA, USA. Mechanical testing: Multiscale Mechanics of Materials Lab, Pennsylvania State University, University Park, PA, USA.
Data accessibility	Repository name: Zenodo.org. Data identification number/DOI: 10008435. Direct URL to data: https://zenodo.org/records/10008435 Instructions for accessing these data: There are no specific restrictions or access controls for the data posted in the repository; all the data are available to access through the direct URL of the data repository or can be found by searching the identification number in the Zenodo platform.
Related research article	Luo, Q., Huang, N., Fu, T., Wang, J., Bartles, D.L., Simpson, T.W. and Beese, A.M., (2023). New insight into the multivariate relationships among process, structure, and properties in laser powder bed fusion AlSi10Mg. Additive Manufacturing, 103804. https://doi.org/10.1016/j.addma.2023.103804

## 1 Value of the Data

• This dataset includes microstructural and property measurements that were obtained through comprehensive experimental investigation of the processing-structure-property (PSP) relationships of laser powder bed fusion (PBF-LB) additively manufactured AlSi10Mg fabricated using a wide range of processing conditions. The microstructure was characterized using X-ray computed tomography (XCT), scanning electron microscope (SEM) imaging, and electron backscatter diffraction (EBSD), and mechanical properties were characterized via mechanical testing and hardness measurements.
• The data provided in this article expand on previous PSP investigations of PBF-LB AlSi10Mg by investigating a wide range of different processing conditions that have not been published in literature for this material.
• The data can be used to support the optimization of PBF-LB AlSi10Mg such that the additively manufactured parts are defect-free with good mechanical properties.
• The data can be used to develop, calibrate, and validate data-driven and Integrated Computational Materials Engineering (ICME) models to better predict PSP relationships in PBF-LB AlSi10Mg.

## 2 Objective

The objective of this dataset is to provide experimentally quantified PSP feature data for AlSi10Mg fabricated using PBF-LB additive manufacturing (AM), which can be used to develop, calibrate, and validate PSP models for this material system.

## 3 Data description

A total of 60 processing sets with different combinations of laser power (60–460 W), scan speed (250–3000 mm/s), and hatch spacing (100 and 150 µm) were intentionally used to probe a wide range of processing regimes, including dense, lack of fusion (LoF), and keyholing. Uniaxial tensile samples, in accordance with ASTM E8/E8M [2], were fabricated using a 3D System ProX DMP 320 PBF-LB AM system machine. As-fabricated samples were direct aged for six hours at 170 °C followed by five hours of furnace cooling in an Argon gas atmosphere. Microstructural characterization, using XCT and SEM imaging, and mechanical testing, though uniaxial tension testing and microhardness testing, were performed to quantify the microstructure and property of the material fabricated using different processing conditions.

The dataset includes eight processing parameters, nine microstructural features, and five mechanical property features, as shown in Table 1. The processing conditions used in this study are given in Table 2, and the statistical mean and standard deviation values of each PSP feature, measured for each processing parameter set, are summarized in Tables 3 through 9. For each PSP feature given in Tables 3 through 9, the experimental testing or characterization methods and the corresponding table index are listed in Table 1.

**Table 1:** Summary of processing, microstructural, and mechanical property features.

Feature type	Experimental methods	Extracted process-structure-property feature	Table index
Processing conditions	Prior fabrication designed processing parameters and descriptors	Laser power P	Table 2
		Scan speed v	
		Layer thickness l	
		Hatch spacing h	
		Linear energy density LED (P/v)	
		Volumetric energy density VED (P/vhl)	
		Modified volumetric energy density MVED (P/√v)	
		Laser power multiplied by scan speed Pv (Pv)	
Microstructural features	Archimedes density measurements	Pore volume fraction (Archimedes porosity)	Table 3
	X-ray computed tomography	Pore volume fraction (XCT porosity)	Table 3
		Pore equivalent diameter	Table 4
		Pore sphericity	Table 4
		Surface roughness	Table 5
	Electron backscatter diffraction	Grain equivalent diameter	Table 6
		Grain circularity	Table 6
		Grain eccentricity	Table 6
	Scanning electron microscope	Cell equivalent diameter	Table 7
Mechanical properties	Hardness indentation	Vickers microhardness	Table 8
	Uniaxial tension	Yield strength	Table 9
		Ultimate tensile strength	Table 9
		Elongation to fracture	Table 9
		Elastic modulus	Table 9

**Table 2:** Processing conditions used to fabricate samples. A constant layer thickness of 60 µm was used to fabricate all samples.

Parameter set		Processing parameters		Processing descriptors			
		Laser power P (W)	Scan speed v (mm/s)	LED (J/m)	VED (J/mm^3^)	MVED (J/√(m/s))	Pv (*W* × *m*/s)
1	Small hatch spacing (*h* = 100 μm)	60	250	0.24	40	4	15
2		160	250	0.64	107	10	40
3		160	800	0.20	33	6	128
4		160	1350	0.12	20	4	216
5		260	250	1.04	173	16	65
6		260	800	0.33	54	9	208
7		260	1350	0.19	32	7	351
8		260	1900	0.14	23	6	494
9		360	250	1.44	240	23	90
10		360	800	0.45	75	13	288
11		360	1350	0.27	44	10	486
12		360	1900	0.19	32	8	684
13		360	2450	0.15	24	7	882
14		360	3000	0.12	20	7	1080
15		460	250	1.84	307	29	115
16		460	800	0.58	96	16	368
17		460	1350	0.34	57	13	621
18		460	1900	0.24	40	11	874
19		460	2450	0.19	31	9	1127
20		460	3000	0.15	26	8	1380
21		110	250	0.44	73	7	28
22		210	250	0.84	140	13	53
23		60	500	0.12	20	3	30
24		110	500	0.22	37	5	55
25		160	500	0.32	53	7	80
26		210	500	0.42	70	9	105
27		260	500	0.52	87	12	130
28		360	500	0.72	120	16	180
29		460	500	0.92	153	21	230
30		110	800	0.14	23	4	88
31		210	800	0.26	44	7	168
32		210	1350	0.16	26	6	284
33	Large hatch spacing (*h* = 150 μm)	60	250	0.24	27	4	15
34		160	250	0.64	71	10	40
35		160	800	0.20	22	6	128
36		260	250	1.04	116	16	65
37		260	800	0.33	36	9	208
38		260	1350	0.19	21	7	351
39		360	250	1.44	160	23	90
40		360	800	0.45	50	13	288
41		360	1350	0.27	30	10	486
42		360	1900	0.19	21	8	684
43		460	250	1.84	204	29	115
44		460	800	0.58	64	16	368
45		460	1350	0.34	38	13	621
46		460	1900	0.24	27	11	874
47		460	2450	0.19	21	9	1127
48		110	250	0.44	49	7	28
49		110	500	0.22	24	5	55
50		160	500	0.32	36	7	80
51		260	500	0.52	58	12	130
52		360	500	0.72	80	16	180
53		460	500	0.92	102	21	230
54		210	250	0.84	93	13	53
55		210	500	0.42	47	9	105
56		210	800	0.26	29	7	168
57		210	1100	0.19	21	6	231
58		260	1100	0.24	26	8	286
59		360	1100	0.33	36	11	396
60		460	1100	0.42	46	14	506

**Table 3:** Porosity calculated using X-ray computed tomography (XCT) and Archimedes density measurements.

Parameter set	XCT porosity (%)		Archimedes porosity (%)	
	Mean	Standard deviation	Mean	Standard deviation
1	0.0446	0.0203	0.9734	0.1651
2	0.2705	0.0194	0.8935	0.3625
3	0.2608	0.0340	1.0274	0.3868
4	0.0694	0.0056	0.6073	0.3417
5	0.0915	0.0062	1.0962	0.2508
6	0.1133	0.0090	1.2439	0.3003
7	0.2466	0.0365	0.5570	0.2662
8	0.0985	0.0076	0.8359	0.3776
9	0.2717	0.0323	1.3683	0.4264
10	0.2165	0.0178	0.6199	0.2252
11	0.2481	0.0214	0.7047	0.2986
12	0.2820	0.0252	1.1584	0.2027
13	2.5762	0.0891	4.8518	0.3163
14	0.2935	0.0212	0.7578	0.2216
15	1.1472	0.0783	0.7701	0.1741
16	3.9892	0.2051	4.0145	0.4364
17	0.1653	0.0136	0.8009	0.4792
18	0.1987	0.0165	0.8195	0.3532
19	0.2065	0.0154	0.6189	0.4358
20	0.1694	0.0125	0.2575	0.6658
21	0.2045	0.0159	0.8811	0.2908
22	0.1770	0.0146	1.0906	0.3024
23	0.1967	0.0168	0.7893	0.3173
24	0.1807	0.0152	1.2608	0.0942
25	0.2477	0.0212	0.9277	0.6833
26	0.1275	0.0111	0.8044	0.2251
27	0.2444	0.0177	1.1278	0.2243
28	0.1963	0.0131	1.1933	0.2091
29	0.1430	0.0099	1.5582	0.5804
30	0.2067	0.0152	0.9318	0.0967
31	0.2239	0.0151	1.0282	0.1103
32	0.6734	0.0335	1.4904	0.2005
33	14.4274	0.4063	16.4258	0.3373
34	0.4051	0.0246	1.1607	0.2266
35	5.4561	0.1614	3.5563	0.2370
36	8.0005	0.1541	6.0473	0.1575
37	0.3139	0.0220	1.3992	0.3915
38	1.9600	0.0730	2.0840	0.4338
39	6.8676	0.1324	5.2693	0.3618
40	4.9747	0.1894	4.4465	0.1060
41	0.1784	0.0137	1.2756	0.3793
42	1.2544	0.0558	2.0912	0.3718
43	6.2806	0.1446	3.9010	0.3442
44	7.2545	0.2078	5.6942	0.0855
45	0.4658	0.0292	1.4451	0.2219
46	0.2388	0.0169	1.2883	0.2570
47	0.8722	0.0480	1.6273	0.1096
48	0.8339	0.0458	3.6216	0.1763
49	10.6293	0.2747	5.6434	0.1344
50	0.9915	0.0438	1.8227	0.1098
51	5.1966	0.2633	3.5954	0.2295
52	7.9129	0.1766	5.9258	0.2371
53	6.5743	0.1688	5.0568	0.0775
54	4.7935	0.2031	3.8082	0.1999
55	0.2770	0.0205	1.2683	0.2776
56	0.3736	0.0258	1.2428	0.1176
57	2.5826	0.0829	2.6287	0.2249
58	0.4387	0.0335	1.2619	0.2387
59	1.1160	0.0694	1.8223	0.3966
60	2.4517	0.0916	3.4679	0.2138

**Table 4:** Pore equivalent diameter and sphericity measured using X-ray computed tomography. The numerical mean (mean) and volume weighted mean (Vol-mean) are presented.

Parameter set	Pore equivalent diameter (µm)			Pore sphericity		
	Mean	Standard deviation	Vol-mean	Mean	Standard deviation	Vol-mean
1	41	12	45	0.77	0.11	0.74
2	63	22	70	0.88	0.08	0.90
3	56	24	66	0.80	0.11	0.79
4	55	19	61	0.82	0.07	0.82
5	54	19	60	0.83	0.07	0.83
6	61	23	69	0.84	0.08	0.85
7	61	24	71	0.84	0.11	0.83
8	55	19	62	0.83	0.08	0.85
9	59	22	67	0.83	0.08	0.83
10	62	23	70	0.87	0.09	0.88
11	63	22	71	0.87	0.09	0.88
12	64	23	71	0.87	0.09	0.89
13	89	73	146	0.82	0.13	0.52
14	63	27	75	0.85	0.09	0.83
15	73	26	82	0.91	0.09	0.93
16	89	32	100	0.92	0.09	0.91
17	58	20	65	0.85	0.08	0.84
18	57	20	64	0.84	0.07	0.85
19	59	21	66	0.85	0.08	0.86
20	56	20	63	0.84	0.07	0.85
21	60	22	68	0.84	0.08	0.86
22	61	22	69	0.84	0.09	0.85
23	62	23	70	0.86	0.08	0.87
24	60	22	68	0.84	0.09	0.83
25	58	20	65	0.83	0.07	0.83
26	56	21	64	0.83	0.08	0.84
27	61	21	69	0.85	0.07	0.85
28	60	22	68	0.85	0.08	0.86
29	54	19	61	0.82	0.07	0.82
30	62	23	70	0.86	0.09	0.89
31	61	22	69	0.86	0.08	0.87
32	72	35	90	0.87	0.09	0.76
33	59	86	309	0.85	0.07	0.37
34	69	30	82	0.87	0.08	0.84
35	95	88	175	0.83	0.14	0.42
36	146	67	173	0.91	0.09	0.89
37	63	22	70	0.89	0.08	0.91
38	85	67	136	0.83	0.12	0.55
39	139	76	175	0.88	0.10	0.88
40	95	35	108	0.92	0.09	0.91
41	57	20	65	0.84	0.08	0.85
42	80	56	119	0.83	0.11	0.62
43	125	70	160	0.90	0.09	0.88
44	115	51	136	0.90	0.10	0.89
45	64	25	74	0.87	0.09	0.89
46	57	20	64	0.84	0.07	0.85
47	71	39	93	0.85	0.10	0.71
48	71	39	168	0.85	0.10	0.71
49	68	81	276	0.87	0.09	0.49
50	76	44	102	0.86	0.09	0.73
51	92	40	108	0.91	0.10	0.90
52	135	69	166	0.91	0.09	0.89
53	119	63	149	0.91	0.09	0.90
54	91	36	105	0.93	0.08	0.92
55	63	22	70	0.87	0.07	0.88
56	66	30	80	0.86	0.09	0.82
57	89	72	147	0.83	0.13	0.51
58	65	27	77	0.86	0.10	0.86
59	74	25	82	0.92	0.09	0.93
60	85	30	95	0.93	0.07	0.92

**Table 5:** Arithmetic average (Ra) and root mean square average (RMS) surface roughness measured using X-ray computed tomography.

Parameter set	Surface roughness (µm)			
	Ra		RMS	
	Mean	Standard deviation	Mean	Standard deviation
1	13.95	2.38	17.96	3.13
2	12.42	2.06	15.71	2.61
3	17.57	2.61	21.68	3.05
4	17.27	2.12	22.16	2.93
5	17.37	1.97	21.50	2.73
6	15.60	3.35	20.09	5.86
7	16.25	4.12	20.72	5.34
8	14.75	1.98	18.76	2.49
9	14.12	3.36	18.48	4.17
10	17.02	4.47	23.15	10.23
11	15.69	1.44	19.96	1.52
12	15.39	1.55	19.71	2.32
13	14.42	1.63	18.62	2.24
14	14.98	1.51	18.96	1.76
15	14.15	1.45	17.83	1.74
16	24.62	12.46	31.39	16.47
17	16.83	2.15	21.01	2.90
18	17.22	3.82	21.44	4.93
19	16.09	2.27	20.84	4.43
20	14.86	1.48	18.76	1.26
21	14.51	1.95	18.21	1.85
22	14.41	2.09	18.89	2.20
23	14.25	3.63	17.94	4.10
24	18.16	4.67	22.54	5.60
25	15.60	1.99	19.21	2.52
26	14.96	2.46	18.86	2.94
27	15.75	1.35	19.31	1.68
28	15.98	2.49	20.14	3.07
29	15.25	1.56	18.87	1.84
30	15.91	2.37	19.37	3.17
31	15.17	2.36	19.98	2.38
32	15.14	2.23	19.25	3.01
33	13.65	2.68	17.57	2.17
34	14.01	2.03	17.29	3.26
35	13.83	1.70	17.61	1.99
36	14.57	3.45	18.48	3.47
37	14.42	1.91	17.79	2.77
38	14.11	1.69	17.49	1.46
39	11.68	1.84	14.63	2.31
40	18.94	5.39	24.06	7.03
41	14.02	1.18	17.03	1.98
42	13.71	1.66	17.07	2.07
43	14.17	1.48	17.09	1.66
44	13.40	1.80	16.57	2.17
45	15.69	2.38	19.55	2.93
46	13.96	1.60	17.76	1.44
47	14.13	1.29	17.92	1.51
48	16.23	3.47	20.17	4.42
49	13.47	0.88	16.80	1.66
50	15.39	1.72	19.68	3.57
51	15.49	1.56	20.07	1.81
52	14.01	1.19	17.94	2.22
53	17.12	4.58	23.72	8.43
54	13.60	2.31	16.77	3.46
55	15.29	1.47	19.30	2.27
56	15.32	1.27	19.64	1.89
57	12.56	2.13	15.97	1.96
58	15.54	2.63	19.43	3.20
59	15.03	1.38	18.70	1.56
60	23.74	11.37	30.01	14.33

**Table 6:** Grain equivalent diameter, circularity, and eccentricity measured from electron backscatter diffraction data for small hatch spacing samples. The numerical mean (mean) and volume weighted mean (Vol-mean) are presented.

Parameter set	Grain equivalent diameter (µm)			Grain circularity			Grain eccentricity		
	Mean	Standard deviation	Vol-mean	Mean	Standard deviation	Vol-mean	Mean	Standard deviation	Vol-mean
1	7.22	6.54	5.75	0.79	0.59	0.39	0.77	0.27	0.90
2	8.67	9.88	7.02	0.87	0.83	0.29	0.79	0.27	0.94
3	8.08	8.89	6.61	0.81	0.64	0.32	0.77	0.28	0.93
4	7.16	7.10	5.88	0.85	0.75	0.36	0.77	0.26	0.91
5	7.25	6.98	5.87	0.82	0.73	0.36	0.78	0.25	0.91
6	7.18	6.83	5.81	0.90	0.83	0.39	0.75	0.28	0.89
7	8.97	9.76	7.06	0.84	0.82	0.30	0.79	0.27	0.94
8	6.71	6.40	5.56	0.86	0.76	0.39	0.76	0.26	0.90
9	9.37	9.90	7.19	0.70	0.44	0.31	0.76	0.30	0.94
10	8.29	9.75	6.89	0.89	0.85	0.28	0.78	0.28	0.94
11	8.37	9.38	6.81	0.89	0.86	0.30	0.78	0.28	0.94
12	7.25	7.30	5.96	0.84	0.75	0.35	0.77	0.26	0.91
13	7.83	7.27	6.11	0.80	0.73	0.38	0.78	0.25	0.90
14	6.99	6.56	5.69	0.86	0.76	0.39	0.76	0.27	0.89
15	8.58	9.97	7.02	0.85	0.82	0.29	0.78	0.28	0.94
16	7.73	8.55	6.43	0.97	0.92	0.31	0.75	0.30	0.93
17	8.07	8.69	6.55	0.89	0.85	0.33	0.77	0.28	0.92
18	8.72	7.64	6.45	0.69	0.41	0.39	0.74	0.29	0.89
19	7.49	7.04	5.95	0.85	0.78	0.38	0.77	0.26	0.90
20	8.41	8.93	6.70	0.87	0.84	0.32	0.78	0.28	0.92
21	9.93	10.85	7.56	0.65	0.36	0.30	0.78	0.26	0.92
22	8.35	9.69	6.89	0.95	0.91	0.29	0.76	0.30	0.93
23	8.36	8.46	6.56	0.84	0.80	0.34	0.79	0.26	0.92
24	10.64	12.77	8.21	0.68	0.43	0.28	0.76	0.29	0.92
25	10.44	13.82	8.43	0.73	0.60	0.24	0.80	0.25	0.94
26	10.63	13.40	8.36	0.65	0.40	0.25	0.79	0.27	0.95
27	9.30	10.57	7.34	0.70	0.53	0.30	0.80	0.24	0.92
28	10.55	11.05	7.76	0.72	0.49	0.32	0.73	0.33	0.91
29	11.43	12.91	8.41	0.68	0.47	0.29	0.75	0.33	0.93
30	9.93	9.48	7.22	0.68	0.45	0.35	0.74	0.32	0.91
31	12.05	12.52	8.45	0.66	0.45	0.30	0.75	0.30	0.90
32	13.82	22.07	11.07	0.72	0.54	0.15	0.75	0.36	0.97

**Table 7:** Sub-grain cell equivalent diameter measured from scanning electron microscope images for small hatch spacing samples.

Parameter set	Cell equivalent diameter (µm)	
	Mean	Standard deviation
1	0.90	0.57
2	1.29	1.02
3	1.01	0.75
4	0.95	0.63
5	1.05	0.75
6	0.98	0.67
7	1.09	0.86
8	0.77	0.39
9	1.05	0.73
10	0.95	0.63
11	0.99	0.68
12	0.98	0.65
13	0.98	0.65
14	0.94	0.62
15	1.12	0.77
16	1.11	0.81
17	1.11	0.84
18	0.92	0.60
19	0.95	0.54
20	0.98	0.63
21	1.09	0.77
22	1.25	0.97
23	0.97	0.61
24	1.01	0.61
25	0.93	0.62
26	1.09	0.75
27	0.94	0.59
28	1.07	0.73
29	0.97	0.64
30	1.13	0.81
31	1.02	0.63
32	0.96	0.63

**Table 8:** Measured Vickers microhardness as a function of parameter set.

Parameter set	Vickers microhardness (HV)	
	Mean	Standard deviation
1	117	4
2	115	5
3	115	5
4	140	8
5	118	1
6	110	5
7	113	8
8	114	7
9	119	6
10	114	6
11	115	4
12	117	4
13	118	3
14	108	4
15	112	6
16	113	7
17	115	8
18	111	7
19	115	3
20	114	7
21	109	6
22	113	7
23	124	5
24	115	10
25	115	6
26	118	9
27	106	3
28	121	6
29	123	6
30	120	5
31	120	9
32	124	8
33	121	8
34	114	8
35	122	7
36	85	13
37	109	6
38	115	8
39	78	15
40	106	5
41	117	7
42	108	5
43	82	19
44	98	5
45	115	6
46	119	7
47	121	8
48	104	2
49	119	12
50	124	10
51	114	8
52	117	14
53	109	6
54	111	7
55	122	5
56	116	8
57	123	9
58	124	9
59	114	5
60	118	7

**Table 9:** Yield strength, ultimate tensile strength, elongation to fracture, and elastic modulus measured using uniaxial tension testing.

Parameter set	Yield strength (MPa)		Ultimate tensile strength (MPa)		Elongation to fracture (%)		Elastic modulus (GPa)	
	Mean	Standard deviation	Mean	Standard deviation	Mean	Standard deviation	Mean	Standard deviation
1	223	4	372	5	3.19	0.14	72	3
2	220	3	385	1	3.54	0.32	68	8
3	192	26	362	15	3.35	0.07	60	4
4	216	3	375	3	3.41	0.15	76	9
5	223	5	375	5	3.19	0.19	75	3
6	214	1	374	5	3.45	0.00	79	7
7	212	0	360	0	3.25	0.00	72	0
8	220	1	370	4	3.41	0.17	70	5
9	220	0	376	5	3.22	0.08	66	0
10	226	9	375	3	3.58	0.15	68	1
11	220	0	376	2	3.58	0.01	70	1
12	223	2	382	1	3.89	0.12	70	2
13	224	4	379	4	3.54	0.13	71	1
14	224	4	381	4	3.82	0.08	70	1
15	222	5	376	5	3.56	0.09	71	3
16	220	4	371	5	3.57	0.03	66	2
17	227	1	381	2	3.58	0.06	67	1
18	219	3	372	6	3.31	0.25	73	4
19	218	2	373	6	3.56	0.22	71	3
20	223	1	381	2	3.71	0.08	70	1
21	228	1	382	7	3.38	0.24	72	1
22	223	0	379	1	3.85	0.08	72	4
23	237	1	385	2	3.25	0.21	77	5
24	228	3	380	7	3.39	0.28	73	2
25	225	0	383	3	3.58	0.09	76	0
26	226	0	384	5	3.69	0.02	76	5
27	226	0	381	0	3.61	0.10	73	1
28	228	3	381	4	3.39	0.13	76	4
29	240	9	388	5	3.56	0.03	68	7
30	226	1	379	3	3.46	0.23	71	1
31	226	5	344	21	2.55	0.37	65	2
32	196	41	258	78	1.35	0.68	67	2
33	150	14	184	22	0.87	0.09	55	5
34	203	14	285	40	1.98	0.70	62	8
35	181	19	252	4	2.38	1.42	60	6
36	161	18	297	49	4.01	0.75	58	9
37	216	10	328	35	2.79	0.71	65	6
38	201	27	298	33	2.82	0.70	58	6
39	164	16	256	15	3.96	1.13	59	7
40	202	23	317	39	3.10	0.26	57	0
41	210	22	329	45	2.76	0.47	63	6
42	208	12	312	12	3.13	0.73	61	7
43	157	19	247	24	4.18	1.53	77	21
44	203	28	330	55	3.90	0.21	58	8
45	185	26	308	37	3.68	0.41	56	1
46	229	5	349	21	3.09	0.45	56	7
47	217	18	308	17	2.16	0.37	65	6
48	184	17	249	20	1.53	0.22	51	3
49	159	28	214	30	1.07	0.30	59	4
50	208	19	309	24	2.53	0.57	56	4
51	177	17	292	25	3.62	0.56	53	2
52	155	11	255	15	4.00	0.19	56	3
53	152	21	257	27	4.52	0.18	57	8
54	188	34	308	46	3.45	0.61	57	4
55	219	13	341	13	2.88	0.56	61	6
56	220	16	325	32	2.41	0.54	60	5
57	211	28	318	38	2.16	0.39	65	5
58	220	14	352	24	2.90	0.34	59	4
59	209	10	325	23	2.96	0.50	57	6
60	186	19	296	18	3.02	0.49	60	4

The statistical mean and standard deviation values of all PSP features as a function of processing parameters, as well as the raw experimental data that were used, are available in the Zenodo data repository. A high-level overview of the data can be found as the “AlSi10Mg PSP feature table” Microsoft Excel spreadsheet in the repository. The raw experimental data for all PSP features can be found as individual files of tabular data (for surface roughness, pores, grain/sub-grain structures, and stress-strain behavior) and SEM/EBSD images (for grains and cells).

## 4 Experimental Design, Materials, and Methods

### 4.1 Process map design

Different combinations of laser power, P, scan speed, v, and hatch spacing, h, were used to probe dense and defect processing regimes. A constant layer thickness, l, of 60 µm was used. Similar to previous work on PBF-LB Ti-6Al-4 V [3,4], a variety of quantitative descriptors were used to define the processing condition, including volumetric energy densities and the melt pool instability factor.

The volumetric energy density, VED, and modified volumetric energy density, MVED, were used to estimate the bounds of the LoF regime, which is caused by incomplete fusion of neighboring tracks, and the keyholing regime, which is caused by excessive energy input. Both VED and MVED metrics quantify the energy during fabrication and are defined as:

(1) $$VED\;=\frac{P}{vhl}$$

(2) $$MVED\;=\frac{\beta }{h\sqrt{4\alpha \phi }}\cdot \frac{P}{\sqrt{v}}=C\left(\frac{P}{\sqrt{v}}\right)$$

where α and β are the laser absorptivity and thermal diffusivity for the powder, respectively, ϕ is the laser spot diameter, and C is a material constant. The difference between VED and MVED is that VED assumes that the laser penetration depth is the same as the layer thickness, which is a constant parameter, while MVED considers the penetration depth as a function of laser energy distribution [5].

In addition to VED and MVED, the melt pool instability factor was used to describe the processing regime for beading-up or balling instability, which is caused by the over-elongation of the melt pool, such that the melt pool breaks up into small droplets due to surface tension. The melt pool instability factor is defined by the aspect ratio of the melt pool, which is related to laser power multiplied by scan speed (Pv), defined as [6], [7], [8]:

(3) $${\left(\frac{L}{W}\right)}^{2}=\frac{\epsilon e}{32\pi \kappa \alpha \Delta T}\cdot \left(Pv\right)=C\left(Pv\right)$$

where L and W are the length and width of the melt pool along the fabrication substrate, respectively, ϵ is the laser absorptivity, κ and α are the conductivity and thermal diffusivity of the powder, respectively, ΔT is the temperature difference between the material's melting point and room temperature, e is Euler's number, and C is a material constant.

### 4.2 Sample fabrication

Tensile samples were fabricated with a 3D Systems ProX DMP 320 PBF-LB system machine (3D System, Inc., United States) using gas atomized LaserForm® AlSi10Mg (A) AlSi10Mg metallic powder (3D System, Inc., United States) with the elemental composition of 9–11 Si, 0.2–0.45 Mg, 0.55 Fe, 0.03 Cu, 0.35 Mn, 0.15 Ti, and balance Al (wt.%) [9]. After fabrication, a direct ageing treatment at 170° for six hours, followed by five hours of furnace cooling, in an Argon gas atmosphere was applied to all samples [10].

All samples were designed with a dog-bone flat geometry according to ASTM E8/E8M [2] with the vertical build direction parallel to the tensile axis. An outer contour was applied using constant processing parameter (275 W, 800 mm/s) to minimize variations in surface roughness between samples. The interior bulk of each sample was fabricated using different processing parameters, which are given in Table 2. A 245° hatch rotation was applied between each subsequent layer. A constant layer thickness of 60 µm was used for all samples.

### 4.3 Internal pore characterization

XCT was used to characterize the internal pore structure of samples using a Phoenix v|tome|x L300 nano/micro-CT (General Electric, Inc., United States) machine with a voltage of 200 kV, current of 50 uA, and a voxel size of 10 µm. Samples were rotated 360° during imaging to capture a total of 1400 2D images with 1 s of exposure time. The 2D images were then reconstructed to reveal the 3D volume information using software [11] (Phoenix datos|x, Waygate Technologies, Inc., United States).

XCT data were analyzed using image processing software, including Amira-Avizo [12] (v. 2020.2, Thermo Fisher Scientific, Inc., United States) and MATLAB [13] (v. R2018b, The MathWorks, Inc., United States). Due to pore detection resolution limits of XCT [14], internal pores with edge lengths smaller than three times the voxel sizes (30 µm) were removed from analysis. Quantitative features were extracted from XCT data, including size and morphological information of each pore (equivalent diameter and sphericity), surface roughness of the gauge region along the vertical build direction (average and RMS surface roughness), and pore volume fraction (XCT porosity). Archimedes density measurements were also performed to measure the porosity of each sample (Archimedes porosity) in accordance with ASTM B962 [15] as a comparison to XCT porosity. For all quantitative features, the statistical mean and standard deviation values were calculated for each processing parameter set.

### 4.4 Grain/sub-grain characterization

Grain and sub-grain solidification cells were characterized through imaging of the undeformed grip of small hatch spacing samples after tensile testing. The grip region was removed from the fractured sample using a low speed cutting saw, mounted into a 1.25-inch epoxy puck using an electro-hydraulic automatic mounting press (TechPress 3, Allied High Tech Products, Inc., United States), and prepared for imaging using a standard metallurgical grinding and polishing procedure with a final polish using 0.05 µm colloidal silica suspension.

EBSD (Apreo 2, ThermoFisher, United States) was used with an accelerating voltage of 10 kV, a step size of 2 µm, and the Aztec software (Oxford Instruments, United Kingdom) to characterize grains from polished surfaces. Samples were then etched using Keller's etchant (1 wt% hydrofluoric acid, 2.5 wt% nitric acid, and 1.5 wt% hydrochloric acid, in de-ionized water) for 30 s to reveal sub-grain solidification cells, which were characterized using SEM (Verios G4 XHR, ThermoFisher, United States) imaging with an accelerating voltage of 10 kV.

EBSD images of grains and SEM images of cells were analyzed using MATLAB [13] to quantify the grain equivalent diameter (according to ASTM E2627 [16]), grain circularity, grain eccentricity, and cell equivalent diameter.

### 4.5 Mechanical testing

Uniaxial tensile tests and Vickers microhardness measurements were performed to measure the mechanical properties of samples. Prior to tensile testing, specimens were painted with black and white speckle patterns to create trackable features for digital image correlation (DIC) to measure fields during tensile loading. An electromechanical load frame (Criterion Model 45, MTS Systems, Inc., United States) with maximum load of 10 kN was used to apply a quasi-static strain rate of 3 × 10^−4^ /s. The gauge region of the samples was imaged using a digital camera (GRAS-50S5M-C, Point Grey Research, Inc., British Columbia) at a rate of 1 Hz using imaging software (VIC-Snap 9, Correlated Solutions, Inc., United States). The surface deformation and strain field were calculated using a DIC analysis software (VIC-2D 6, Correlated Solutions, Inc., United States). A cubic B spline interpolation algorithm, with a subset size of 29 pixels and a step size of 7 pixels, where the pixel size ranged from 15.7 to 25.9 µm, and a 24 mm vertical virtual extensometer were used to measure the axial strain. The ultimate tensile strength, yield strength, elongation to fracture, and elastic modulus were extracted from the stress-strain response of each sample. Three duplicate samples were tested for each processing parameter set to obtain statistical mean and standard deviation values of the mechanical properties.

Vickers microhardness measurements (LM 110AT, LECO, Inc., United States) were performed according to ASTM E384 [17]. A total of 10 indents were made at dense locations within each sample using a load of 100 gf and a dwell time of 10 s.

## Limitations

Flat tensile samples, all aligned with the vertical build direction, with constant thickness were studied to avoid additional impacts from sample geometry on part microstructure or mechanical properties. The characterization of grain/sub-grain features was only conducted on small hatch spacing samples. While 180 tensile samples were fabricated (3 duplicates for each processing parameter set), two samples failed during installation in the mechanical test frame, so mechanical test results from only 178 samples are included.

## Ethics Statements

No human subjects, animal experiments and data collected from social media platforms were involved in this work.

## CRediT authorship contribution statement

**Qixiang Luo:** Writing – original draft, Visualization, Software, Methodology, Investigation, Formal analysis, Data curation. **Nancy Huang:** Writing – review & editing, Investigation, Formal analysis. **Tianyi Fu:** Investigation, Formal analysis. **Jinying Wang:** Investigation, Formal analysis. **Dean L. Bartles:** Writing – review & editing. **Timothy W. Simpson:** Writing – review & editing, Funding acquisition. **Allison M. Beese:** Writing – review & editing, Supervision, Resources, Project administration, Methodology, Funding acquisition, Conceptualization.

## Data Availability

Processing, microstructure, and mechanical property dataset for AlSi10Mg fabricated by laser powder bed fusion additive manufacturing (Original data) (Zenodo)
